# Two Good Papers

**Published:** 1894-01

**Authors:** 


### TWO GOOD PAPERS.

It is not our custom to call special attention to papers published in this journal, but we fear that oftentimes our readers lay the numbers, as they appear, aside, for the leisure to read certain articles, but this period never comes, and the good things contained therein are lost.

We therefore hope that no one will defer reading two of the articles in this number, the one—Professor Peirce's paper—for its possible scientific value and generally excellent and altogether interesting statement of supposed facts connected with a rather overwritten subject. The other on "Specialism," by Rev. Reuen Thomas, is an address worthy of careful perusal. The genial tone and scholarly thought pervading it gives to it a value not only to our profession but to all workers in narrow scientific fields. Its broad catholic spirit is refreshing in these days of scientific platitudes and profound dulness, the necessary outgrowth of a limitation of vision.

